# Attitudes toward Death among Health Care Professionals in the Balkan Region

**DOI:** 10.3390/curroncol31060255

**Published:** 2024-06-16

**Authors:** Tomi Kovacevic, Bojan Zaric, Jelena Djekic Malbasa, Darijo Bokan, Borislava Nikolin, Daliborka Bursac, Petar Simurdic, Vladimir Stojsic, Goran Stojanovic, Dragana Maric

**Affiliations:** 1 University of Novi Sad, Faculty of Medicine, 21102 Novi Sad, Serbia; bojan.zaric@mf.uns.ac.rs (B.Z.); jelena.djekic-malbasa@mf.uns.ac.rs (J.D.M.); darijo.bokan@mf.uns.ac.rs (D.B.); borislava.nikolin@mf.uns.ac.rs (B.N.); daliborka.bursac@mf.uns.ac.rs (D.B.); petar.simurdic@institut.rs (P.S.); vladimir.stojsic@mf.uns.ac.rs (V.S.); 2 Institute for Pulmonary Diseases of Vojvodina, 21204 Sremska Kamenica, Serbia; goran.stojanovic@institut.rs; 3 Clinical Center of Vojvodina, 21137 Novi Sad, Serbia; 4 Oncology Institute of Vojvodina, 21204 Sremska Kamenica, Serbia; 5 Univerity Business Academy in Novi Sad, Faculty of Pharmacy, 21101 Novi Sad, Serbia; 6 University of Belgrade, Faculty of Medicine, 11000 Belgrade, Serbia; dragana.maric@med.bg.ac.rs; 7 University Clinical Center of Serbia, 11000 Belgrade, Serbia

**Keywords:** death attitude, palliative medicine, fear of death, physicians, nurses, attitudes toward death, death and dying

## Abstract

*Background and Objectives*: Death is an unavoidable experience in any person’s life and affects not only the dying person but also their caregivers. The dying process has been displaced from homes to health care facilities in the majority of cases. Facing death and dying has become an everyday life of health care professionals (HCP), especially in palliative care (PC) settings. This study aimed to investigate the death attitudes among HCPs in Serbia. *Materials and Methods*: The Serbian version of the Death Attitude Profile-Revised (DAP-RSp) was used as a measurement instrument. *Results*: The average age of the 180 included participants was 42.2 ± 9.9 years; the majority were females (70.0%), with more than 10 years of working experience (73.0%), physicians (70.0%) and those working in a non-oncological (non-ONC) field (57.78%). The mean total score of DAP-RSp was 124.80 ± 22.44. The highest mean score was observed in the neutral acceptance dimension (NA) (5.82 ± 0.90) and lowest in the Escape acceptance (EA) (2.57 ± 1.21). Higher negative death attitudes were reported among nurses compared to physicians (*p* = 0.002). Statistically significant differences were observed in the fear of death (FD) and death avoidance (DA) domains, favoring PC specialists and oncologists (*p* = 0.004; *p* = 0.015). Physicians working in Oncology (ONC) showed lower FD values (*p* = 0.001) compared to non-ONC departments. *Conclusions*: Attitudes toward death among HCPs are of great importance for the well-being of both HCPs and patients. Negative attitudes can lead to deficient care. The fear of death is highly represented among Serbian HCPs working in non-ONC fields, including both nurses and physicians. This study emphasizes the need for further research to comprehensively explore and understand HCPs’ attitudes toward death. This research highlights the need for the development of an educational curriculum across all levels of medical education, aimed at overcoming the fear of death and enhancing coping strategies, which will improve the care for patients diagnosed with terminal illnesses.

## 1. Introduction

As a part of a life cycle, death is an unavoidable experience of any person’s life. Historically, death is one of the greatest mysteries and still remains the bitter reality with which every one of us needs to cope. Despite the fact that death is a natural process, emotions and attitudes toward death are different depending on culture, education, social aspects, country of origin, spirituality and many other individual factors [1]. Each person experiences death uniquely, and there is no single way to experience it. Confronting death and dying as a powerful irreversible end triggers emotional reactions, not only the overwhelming feelings of loss but also awareness and fear of one’s own death. Dying, in the end, is the process of awareness and acceptance of inevitability. In order to develop a coping mechanism and to enhance a positive approach through trainings and education, it is of great importance to address and explore attitudes toward death and dying [2,3,4,5].

In the last century, the trajectory of the dying process changed. Civilizational and technical progress and, consequently, achievements in medicine have led to a prolonged life span. Nowadays, people are healthier, live longer and die later. On the other hand, the dying process, often significantly prolonged, has been displaced from the traditional setting of dying persons’ homes to health care institutions [6]. As a physical event, it affects not only the dying person but also has a psychological impact on their caregivers, especially health care professionals (HPCs) [7,8]. Facing death and dying has become an everyday life of HCPs, particularly of those working in institutions caring for patients with cancer and in palliative care settings. This constant exposure gives rise to a mixture of intense emotions, intensifying the fear of death on both conscious and unconscious levels [9].

As an existential experience, death influences every aspect of a person’s life, behavior and functioning: “Often experienced, it may also result from unworked everyday matters or incorrect beliefs about oneself, death, and life” [9]. Constant caring for dying people and their suffering inevitably raises questions about the finitude of one’s own life [9]. The fear of death, described as “apprehension, worry or fear related to one’s own death or dying” by the North American Nursing Diagnosis Association (NANDA), represents one of the deeply ingrained basic human instincts. Essentially, it reflects a struggle for one’s own life on a subconscious level. Passive and indifferent behavior toward death is extremely rare [7,10]. Simply put, fear is an emotional reaction to objective danger, recognized as a defense mechanism for survival, and it is considered a reaction without signs of pathology. The fear of death (thanatophobia) includes the fear of one’s own death, the fear of the death process, and the demise of significant others. In order to better understand this phenomenon, it is important to distinguish between the fear of death as an emotional reflex and thinking about death as a contemplation of a distant death. Thinking about death is a cognitive action aimed at the consciousness of reality, and death anxiety is a multidimensional cognitive, emotional and experimental construct [7,11].

Attitude toward death is “an evaluative and relatively stable internal psychological tendency held by an individual in response to death”, and represents personal feelings about the death (of self and others), and it may be influenced by adopted life philosophy, religion, society, culture and many other individual causes [12,13,14,15]. Death attitudes are important in one’s life because they link death and dying with acceptance of an irreversible and final natural process of life. These attitudes are conventionally differentiated as positive (accepting) and negative (avoidance) [1,16,17]. Attitudes towards death change over time with the great influence of culture, sex, education, country, historical period and religion, as well as due to personal mental imagery and the idea of death [1,18]. The questions and conversations about death and dying are most commonly raised in terms of the time of closeness to large numbers of deaths, e.g., wars and pandemics such as COVID-19 [19,20,21]. 

In terms of research, death attitudes have been of interest for a long time. Early studies measuring attitudes toward death were influenced by social aversion and used the one-dimensional scale focused on negative attitudes (fear, avoidance and denial). It was shown that the death attitude is more complex, as it combines not only negative but also positive feelings. The early 1990s saw the breakout made by Wong et al. proposing a model of death attitudes consisting of five components—fear of death (FD), death avoidance (DA), neutral acceptance (NA), approach acceptance (AA) and escape acceptance (EA)—which was widely used in many studies measuring attitudes toward death. Later on, the original model was revised, and the Death Attitude Profile-Revised was made as an instrument with good reliability and validity and became one of the most frequently used questionnaires for assessing attitudes toward death [12,16,22,23,24]. This scale, to the authors’ knowledge, has already been validated (besides the English version) for use in Brazil, Poland, Germany, Turkey, China, Iran, Japan, Jordan, Portugal, Greece, Spain, Romania, Finland and Serbia [16,24,25,26,27,28,29,30].

The importance of HCP attitudes toward death has been recognized in the past few decades. The provision of good quality care for patients, especially in palliative care settings, is one of the imperatives in medicine that still has not been achieved.

Given that health care institutions serve as the primary location of death in contemporary society, the issues surrounding death and dying have evolved into a clinical concern, particularly within the context of acute health care settings. Moreover, the apprehension of death not only contributes to burnout syndrome but also gives rise to various psychological and related physical challenges. In light of these considerations, the heightened focus on this topic comes as no surprise [11,31]. Each HCP has an individual experience of death, with the dying process evoking unique, individualized emotions. These emotions and attitudes toward death significantly shape the subsequent care they provide. According to some authors, it is perceived that modern medicine is extremely powerful in defeating death, and therefore HCPs are struggling to accept the inevitability of death, which is unequivocally specific for high-income countries with a number of available treatment options [6]. Despite the progress in medicine and the fact that people now live longer, death still occurs and is a part of an HCP’s life even more than before [6]. The aging population (predominantly in Western countries) increases the problem even more, and in the future, HCPs will face death on a daily basis in their regular practices. Hence, emotional management skills and coping with death techniques are some of the key skills HCPs need to acquire in order to provide good medical care, since death and dying have a direct impact both on the medical care they provide and on their personal well-being [7,17]. In recent years, the need for a better understanding of attitudes toward death in HCPs and the importance of improving the acceptance of death and dying have gained prominence, primarily due to better care planning [32]. More profound knowledge of the attitudes toward death is the first step in enhancing training, which is crucial for creating a theoretical framework and developing appropriate curricula to address the existing gaps at all levels of HCP education. This is pivotal for the future knowledge and skills of HCPs, who are often exposed to dying people. It will enable them to enhance their psychological well-being and provide adequate care.

The estimated life expectancy at birth in most Balkan countries is lower compared to Western European countries, ranging from 74.8 years in North Macedonia to 78.6 years in Croatia (the EU average is 81 years) [33,34].

Also, the strong impact of the aging population in Balkan countries was observed. According to estimations for 2040, the percentage of the population aged 60 and more will be around 30%, and in some countries like Serbia and Croatia, it will be around 35% [33].

In the group of ten leading causes of premature death in Balkan countries, the highest rank among non-communicable diseases (NCD) was reported for ischemic heart disease and stroke, malignant neoplasms, particularly lung cancer and Alzheimer’s disease [35].

According to WHO’s European Health Information Gateway data, the burden of NCD and the aging population in the Western Balkan is a discrepancy with the level of health system development. Given the fact that the number of professionally active physicians and nurses is lower compared to Western European countries (physicians—23.3 (Bosnia and Herzegovina) and 29.4 (Serbia) vs. 30.6 (Ireland)—58.8 (Norway); nurses 9.8 (Serbia)—60.8 (Bosnia and Herzegovina) vs. 63.1 (Spain) and 216.5 (Norway) per 10,000 inhabitants), the topic of death and dying is even more significant [33].

The level of development and integration of palliative care (PC) in the Western Balkan, presented as the number of specialized PC services per 100,000 inhabitants, is significantly lower than the EAPC recommendation (2/100,000), and lower in the Western Balkan compared to other European countries (in the range from 0.0 to 0.8/100,000) [34].

Based on highlighted data, the aim of this research was to determine attitudes toward death among HCPs who manage the care of patients with incurable diseases. To the authors’ knowledge, this is the first study conducted in the Western Balkan region, with the exception of Serbia, where the Serbian version of the Death Attitudes Profile-Revised (DAP-RSp) was validated by Maric et al. in the population of medical students [24].

## 2. Materials and Methods

This research was conducted during the First Palliative Medicine Conference of Serbia held in Novi Sad in October 2023. After signing the Informed Consent Form (ICF), the participants answered the question on DAP-RSp together with the questions on demographic data: age, gender, place of residence before graduation, profession, place of work, medical field of employment and working experience. Participation was voluntary and anonymous. The exclusion criterion was the rejection of participation in the survey and uncompleted questionnaires. For the main sample, prior to data collection, a power analysis concerning sample size (one sample means, two-tailed) was performed by G-power (Ver 3.1) effect size 0.8, α = 0.01, resulting in a suggested size N = 138.

The statistical analysis was performed using the SPSS 26.0 (IBM Corporation, Armonk, NY, USA). The categorical variables were presented by numbers and percentages, while the continuous variables were presented by means ± standard deviations (SD). A nonparametric independent two-sample *t*-test was used to compare the means between two groups (the Mann–Whitney U-test), while the Kruskal–Wallis test was used to compare the means for more than two groups. 

## 3. Results

Out of 311 conference participants, 228 took part in this research. All the participants with missing data in their questionnaires were excluded from further analyses. Finally, 180 eligible participants with completed all questionnaire items were included in the study. The majority of the respondents were female (70.0%). The average age of study participants was 42.2 ± 9.9 years, without significant differences in average age distribution between females and males (42.7 ± 9.6 vs. 40.81 ± 10.6, respectively) (*p* = 0.234). Regarding previous secondary education, distribution was equal for medical school vs. other (50:50%). The majority of participants had more than ten years of working experience (73.0%) and worked in the field of pulmonology (28.9%). Most of the study participants were physicians (70.0%). In terms of specialization, Oncology and Palliative Medicine specialists accounted for 23.4%. A great majority of the study sample reported close experience with death (88.9%), as shown in Table 1.

The mean total score of DAP-RSp is 124.80 ± 22.44. The highest mean score was observed for the neutral acceptance subscale (5.82 ± 0.90) followed by the death avoidance subscale (4.26 ± 1.51), while the lowest mean score was for the escape acceptance dimension (2.57 ± 1.21).

The reliability analysis showed that DAP-RSp demonstrated good internal consistency, Cronbach’s alpha coefficient for the whole DAP-RSp was 0.81 and higher than 0.70 in all domains except for the NA scale (0.601), as shown in Table 2.

According to tests of normality, only FD and negative death attitudes variables were normally distributed (*p* = 0.051 and *p* = 0.056, Shapiro–Wilk test). The scores of DAP-RSp subscales for all study participants did not show differences according to age groups, gender, prior experience with death or working experience. Significant differences in some of the DAP-RSp subscales were observed for the previous secondary school, field of work and profession. Study participants with finished medical school showed significantly higher mean values in the DA subscale compared to participants with other secondary education (4.533 ± 1.394) vs. (3.996 ± 1.581), (*p* = 0.020). According to the working field (Oncology vs. non-Oncology), participants showed significant differences in FD, AA and NA dimensions of the DAP-RSp subscale. Higher mean values in FD subscale were observed for non-ONC participants (4.123 vs. 3.285; *p* ˂ 0.001), while in AA (3.738 vs. 3.363; *p* = 0.046) and NA domain (6.042 vs. 5.665; *p* = 0.031), higher values were observed among those in ONC field of work. Significantly higher mean negative death attitudes values were reported among participants working in non-ONC units, while positive death attitudes were significantly higher among those in the ONC field of work. According to the profession, significantly higher mean values in FD, DA and AA dimensions of the DAP-RSp subscale were reported among nurses compared to physicians. FD was significantly higher among nurses compared to physicians (*p* = 0.034). The mean score values in the DA (5.021 vs. 4.000; *p* ˂ 0.001) and AA (3.9509 vs. 3.3945, *p* = 0.031) subscale were also significantly higher among nurses compared to physicians. Also, higher negative death attitudes were reported among nurses compared to physicians (*p* = 0.002), as shown in Table 3.

Statistically significant differences among physician responses to the DAP-RSp subscale were observed by gender, field of work and physician’s specialization. Death avoidance mean value was significantly higher for females compared to males (4.2227 ± 1.53321 vs. 3.4947 ± 1.29593), as well as the median value (4.0032 vs. 3.6000); (*p* = 0.011). Statistically significant differences regarding the physician field of work were observed in mean values of the FD domain and negative death attitudes. The physicians working in ONC departments compared to non-ONC departments showed lower fear of death (3.128 vs. 4.059) (*p* = 0.001) and lower negative death attitudes (3.536 vs. 3.186) (*p* = 0.018). Statistically significant differences regarding the specialization were observed in mean values of FD and DA domains and negative death attitudes. Mean values in the FD subscale were significantly lower among Oncology and Palliative Medicine specialists (*p* = 0.004). Mean values of DA scores were significantly lower for Oncology and Palliative Medicine specialists compared to other specializations (*p* = 0.015). Negative death attitudes were much lower among the oncologists compared to other specializations (*p* = 0.001), as shown in Table 4.

## 4. Discussion

The significance of attitudes toward death among HCPs is indisputable, aiming to foster a better understanding with the goal of improving the well-being of all involved parties—those providing care and those experiencing suffering. Serbia is a country with a notably aging population. According to the latest Census (2022), the average age in the Serbian population was 43.85 years (Census 2011—42.2 years), and the proportion of inhabitants older than 65 was 22.09%, which is an increase of almost 5% compared to the 2011 Census 2011 [36,37]. The life expectancy in the Republic of Serbia in 2022 is 75.6 years (73.0 years for men and 78.1 years for women). The most common causes of death are cardiovascular diseases, followed by malignant diseases (47.3% and 18.5%, respectively) [38]. Among various cancers exhibiting rising trends, lung cancer ranks as the second most common cancer in women (10.5%) and holds the first place in men (21.3%), accounting for 16.2% of all newly diagnosed cancers [39]. All this indicates an inevitable rise in the number of deaths in the future. As dying has shifted from homes to health care institutions in most parts of the world, there has been an increasingly frequent occurrence of death experiences in the lives of HCPs [6].

Comparing the results of our research with the results from the Serbian DAP-Rs validation study on medical students, we observed some differences. HCPs working in the field of Palliative Medicine and Oncology, compared to medical students, showed significantly lower mean values for FD (*p* = 0.00) and higher for NA domain (*p* = 0.00) [24]. Similar results were obtained in this research when comparing physicians specialized in the field of Palliative Medicine and Oncology and those with other specializations. Likewise, when comparing HCPs working in the field of Palliative Care/Oncology and those from the non-oncological field, similar results were derived, too. Regarding specialization, a statistically significant difference was observed in the values of the FD domain, favoring the ONC group (*p* = 0.004). In relation to the field of work, there was a statistically significant difference between ONC and non-ONC groups in FD domains, with higher values in the group of HPCs working in the non-oncological field (*p* = 0.001). Another statistically significant finding was observed in the DA domain concerning a physician’s specialization, where mean values were lower in the ONC group (*p* = 0.025). In both cases, considering the field of HPCs’ work and specialization, there was a statistically significant difference in negative death attitudes between those groups (*p* = 0.028; *p* = 0.001). The task of nursing is to take care of the patients; they spend more time with patients and often accompany the process of the patient’s death. Death is also a natural phenomenon and frequent in the lives of HCPs providing care for patients with terminal illnesses. All of these may lead to less negative attitudes towards death and dying. Although not statistically significant, this research showed differences in mean scores in positive and negative death attitudes between groups favoring HCPs with close experience with death. The mean score in negative attitudes was higher in the group of HCPs without close experience with death (4.225 ± 1.030 vs. 3.945 ± 1.239).

Based on its definition as “an approach that improves the quality of life of patients (adults and children) and their families who are facing problems associated with life-threatening illness, it prevents and relieves suffering through the early identification, correct assessment and treatment of pain and other problems, whether physical, psychosocial or spiritual” (WHO, 2002 [40]), palliative care accepts the end of life and death as a natural process. Therefore, basic education in palliative care is significant for HCPs, especially those working in an oncological field. The need for palliative care is increasing worldwide, not only due to the aging of the population but also because of the rise in patients diagnosed with malignant and other non-communicable diseases. According to WHO, “By 2060 the need for palliative care at the end of life is expected to double” (WHO, 2020). Unfortunately, HCPs worldwide have little or no education in either basic or specialized palliative care, despite the existing curricula [40]. It could be concluded that their attitudes are based on individual experiences and personal conceptual ideas, which raises new questions and avenues for research. In Serbia, palliative care is not fully integrated into either curricula or medical practice, and the skills necessary for the care of patients diagnosed with terminal illnesses are insufficient [6]. The findings in this research, together with the recognition that fear of death may be one of the reasons for inadequate and insufficient care of dying people, additionally highlight the need for a deeper understanding of death attitudes among HCPs and the development of appropriate education curricula at all levels.

It was shown that attitudes toward death are influenced by country, education, gender, culture and religion [1]. Regarding the negative death attitudes domain results, the studies worldwide show that mean values of FD were higher than values of DA in Germany, Portugal, Jordan, Spain, Poland, USA, Turkey and Brazil [3,16,17,18,25,26,41,42,43]. Moving to the east, the results from China show that the greater value in this domain was found in DA with the exception of the research made after the outbreak of COVID-19, where the values of FA and DA were similar [1,12,44]. Looking at our results and observing negative death attitudes domains, we found higher values for DA in the observed population. However, diving deeper into subgroups of physicians, we found out that mean values in the FD subscale were significantly lower among Oncology and Palliative Medicine specialists (*p* = 0.004), along with higher DA scores (*p* = 0.015). Also, the DA mean value was significantly higher for females compared to males (*p* = 0.011), and the results of physicians working in ONC compared to non-ONC departments showed lower FD (*p* = 0.018). Regarding the profession, significantly higher mean values in FD and DA dimensions of the DAP-R subscale were reported among nurses compared to physicians. FD was significantly higher among nurses compared to physicians (*p* = 0.034), and mean score values in DA were also significantly higher among nurses compared to physicians (*p* ˂ 0.001). A higher DA attitude was observed for participants who had previously finished secondary medical school (*p* = 0.020).

Regarding positive death attitudes, the majority of research showed the highest values in NA dimensions, with the exception of those from Portugal, Jordan and Poland [1,3,7,12,16,17,18,25,26,41,42,43,44]. These results are in coordination with the observed Serbian population in our study with some exceptions. In AA and NA domains, higher values were observed among those in the ONC field of work (*p* = 0.046, *p* = 0.031, respectively), and values of AA dimension in subscale according to profession were significantly higher among nurses compared to physicians (*p* = 0.031).

There is no doubt that heightened negative attitudes toward death and grief resulting from patient deaths can contribute to burnout and many other psychological and physical issues. Conversely, an increased competence in dealing with death enhances the quality of life and job satisfaction of HCPs [31,45,46,47,48]. Working in palliative care and providing care for dying people carries the risk of burnout. However, it has been reported that HCPs working in this field express a high level of job satisfaction, and the incidence of burnout among these professionals is lower compared to those in other specialties [47,48,49,50,51,52]. The consequences of dissatisfaction and burnout among HCPs are multidimensional. First of all, there is a detrimental impact on the well-being of HCPs, which may lead to a decline in the delivery of high-quality patient care. At an organizational level, job dissatisfaction may result in frequent sick leaves and change of jobs. These findings emphasize the need for a better understanding of HCPs’ attitudes toward death, as they possess the potential to influence not only the entire health care system but also society as a whole.

The results of this research, including the incidence trend of terminal illnesses and the aging population in the Balkan region, indicate the necessity of urgent actions and further research on this topic.

The lack of PC education and training opportunities in Palliative Medicine has been noticed in Balkan countries. Although medical school teaching PC is nowadays present in the undergraduate curricula with variation in the number of classes and practice in most Balkan countries (except in Montenegro, Croatia and BIH), nursing school teaching PC is still missing in Croatia, Montenegro, Turkey, Serbia and Bosnia and Herzegovina [34]. The development of educational trainings and courses for all HCPs managing patients with incurable diseases with the aim to improve coping with death could be the first step in overcoming the development of psychological distress. Further on, it is of great importance to develop tailored educational programs about coping with death and dying for HCPs on all levels of education and for all professions in regular educational curricula, e.g., medical schools and residency programs.

This study has several limitations. All of the participants in this study are involved in the care and management of patients with diagnosed incurable diseases and the results can not represent the whole HCP society, which limits generalizability. In addition, 72% of the sample of study participants was from Serbia, and therefore detailed analysis between countries of origin (Serbia, Montenegro, Bosnia and Herzegovina, Croatia and Northern Macedonia) could not be performed. Given the diversity of the sample, there is a potential selection bias due to the specificity of the population; however, the authors believe that the effect of such bias could not significantly alter the results.

## 5. Conclusions

Health care professionals’ attitudes toward death play a crucial role in the well-being of both professionals and dying patients. Negative death attitudes affect all parties involved, causing the suffering of HCPs and patients alike. The fear of death is notably higher in the Serbian population of HCPs working in the non-oncological field, including both nurses and physicians. There is a lack of education at all levels within the medical field. The findings of this study underscore the necessity for further research to explore and comprehend HCPs’ attitudes and feelings toward death. This should lead to the development of an educational curriculum aimed at overcoming the fear of death and enhancing coping strategies. Training and education aimed at minimizing the fear of death and improving the ability to cope with death should be one of the focuses for all health care professionals and not only for palliative care. Given the aging trend in Serbia and the whole Balkan region and the rising incidence of non-curable diseases, such a curriculum should be part of all levels of medical education.

## Figures and Tables

**Table 1:** Socio-demographic features of study participants.

**Age (years (mean) ± SD)**		42.2 ± 9.9
		No. (%)
**Gender**	Male	54 (30.0)
	Female	126 (70.0)
**Previous secondary school**	Medical	90 (50.0)
	Other	90 (50.0)
**Profession**	Nurses	38 (21.1)
	Physicians	126 (70.0)
	Other	16 (8.9)
**Physicians by specialization**	Neurology	22 (12.2)
	Pulmonology	16 (8.9)
	Oncology	28 (15.6)
	Palliative Medicine	14 (7.8)
	Other	46 (25.5)
**Working experience**	≤10 years	48 (26.7)
	>10 years	132 (73.3)
**Field of work**	Neurology	20 (11.1)
	Pulmonology	52 (28.9)
	Other	32 (17.8)
	Oncology	40 (22.2)
	Palliative Care	36 (20.0)
**Close experience with death**	Yes	160 (88.9)
	No	20 (11.1)

**Table 2:** Frequency analysis.

Death Attitude Dimension	Items	Mean Score	Kurtosis	Skewness	Cronbach Alpha
Fear of death (FD)	1, 2, 7, 18, 20, 21, 32	3.769 ± 1.390	−0.515	0.048	0.825
Death avoidance (DA)	3, 10, 12, 19, 26	4.264 ± 1.510	−0.830	0.007	0.800
Natural acceptance (NA)	6, 14, 17, 24, 30	5.824 ± 0.903	5.196	−1.717	0.601
Approach acceptance (AA)	4, 8, 13, 15, 16, 22, 25, 27, 28, 31	3.521 ± 1.407	−0.954	0.011	0.912
Escape acceptance (EA)	5, 9, 11, 23, 29	2.575 ± 1.210	−0.724	0.462	0.723
Positive death attitudes (NA, AA, EA)		3.863 ± 0.859	−0.966	0.202	0.831
Negative death attitudes (FD, DA)		3.976 ± 1.218	−0.389	−0.274	0.846
Total score	1–32	124.800 ± 22.440	−0.647	−0.158	0.811

**Table 3:** DAP-RSp subscales (previously finished secondary school, field of work and profession).

Death Attitude Dimension		FD (mean ± SD)	DA (mean ± SD)	NA (mean ± SD)	AA (mean ± SD)	EA (mean ± SD)	Negative Death Attitudes (mean ± SD)	Positive Death Attitudes (mean ± SD)
Previous Secondary School	Other school (n = 90)	3.711 ± 1.454	3.996 ± 1.581	5.858 ± 0.697	3.535 ± 1.458	2.488 ± 1.340	3.829 ± 1.337	3.858 ± 0.788
	Medical school (n = 90)	3.827 ± 1.329	4.533 ± 1.394	5.791 ± 1.073	3.508 ± 1.362	2.662 ± 1.064	4.123 ± 1.074	3.829 ± 1.337
	*p*-value	0.81	0.02	0.584	0.982	0.127	0.088	0.784
Field of Work	Palliative and Oncology (n = 76)	3.285 ± 1.262	4.332 ± 1.642	6.042 ± 0.606	3.738 ± 0.434	2.709 ± 1.225	3.722 ± 1.238	4.061 ± 0.909
	Non-ONC (n = 104)	4.123 ± 1.378	4.215 ± 1.413	5.665 ± 0.441	3.363 ± 1.373	2.477 ± 1.195	4.163 ± 1.174	3.717 ± 0.795
	*p*-value	**0.000**	0.634	**0.031**	**0.046**	0.2	**0.008**	**0.014**
Profession	Physicians (n = 126)	3.616 ± 1.511	4.003 ± 1.498	5.943 ± 0.728	3.394 ± 1.355	2.580 ± 1.225	3.778 ± 1.271	3.831 ± 0.857
	Nurses (n = 38)	4.097 ± 1.102	5.021 ± 1.449	5.537 ± 1.322	3.951 ± 1.292	2.663 ± 1.098	4.483 ± 1.018	4.025 ± 0.827
	*p*-value	**0.034**	**0.000**	0.158	**0.031**	0.553	**0.002**	0.175

**Table 4:** DAP-RSp subscale for physicians (gender, field of work and specialization).

	Death Attitude Dimension	FD (mean ± SD)	DA (mean ± SD)	NA (mean ± SD)	AA (mean ± SD)	EA (mean ± SD)	Negative Death Attitudes (mean ± SD)	Positive Death Attitudes (mean ± SD)
Gender	Male (n = 38)	3.428 ± 1.525	3.495 ± 1.296	5.695 ± 0.878	3.468 ± 1.376	2.663 ± 1.244	3.456 ± 1.209	3.824 ± 0.807
	Female (n = 88)	3.697 ± 0.506	4.223 ± 1.533	6.050 ± 0.629	3.363 ± 1.353	2.544 ± 1.222	3.917 ± 1.278	3.834 ± 0.883
	*p*-value	0.302	0.011	0.05	0.625	0.516	0.079	0.782
Field of Work	Palliative and Oncology (n=60)	3.128 ± 1.244	4.107 ± 1.652	6.087 ± 0.613	3.623 ± 1.532	2.625 ± 1.231	3.536 ± 1.233	3.995 ± 0.954
	Non-ONC (n = 66)	4.059 ± 1.602	3.909 ±1.349	5.812 ± 0.801	3.186 ± 1.144	2.539 ± 1.227	3.999 ± 1.274	3.682 ± 0.735
	*p*-value	0.001	0.531	0.074	0.054	0.769	0.018	0.091
Specialization	Palliative and Oncology (n = 42)	3.102 ± 1.083	3.562 ± 1.239	6.076 ± 0.705	3.428 ± 1.544	2.474 ± 0.218	3.294 ± 0.977	3.859 ± 0.886
	Non-ONC (n = 84)	3.873 ± 1.630	4.224 ± 1.573	5.876 ± 0.735	3.377 ± 1.259	2.633 ± 1.232	4.021 ± 1.335	3.817 ± 0.848
	*p*-value	0.004	0.015	0.103	0.694	0.52	0.001	0.686

## Data Availability

The data presented in this study are available on request from the corresponding author due to legal reasons.
